# Spatio-Temporal Differentiation and Sociality in Spiders

**DOI:** 10.1371/journal.pone.0034592

**Published:** 2012-04-24

**Authors:** Jessica Purcell, João Vasconcellos-Neto, Marcelo O. Gonzaga, Jeffrey A. Fletcher, Leticia Avilés

**Affiliations:** 1 Department of Zoology, University of British Columbia, Vancouver, British Columbia, Canada; 2 Department of Ecology and Evolution, University of Lausanne, Bâtiment Biophore, Lausanne, Switzerland; 3 Department de Zoologia, Instituto de Biologia, Universidade Estadual de Campinas, Campinas, São Paolo, Brazil; 4 Instituto de Biologia, Universidade Federal de Uberlandia, Uberlandia, Minas Gerais, Brazil; University of Western Ontario, Canada

## Abstract

Species that differ in their social system, and thus in traits such as group size and dispersal timing, may differ in their use of resources along spatial, temporal, or dietary dimensions. The role of sociality in creating differences in habitat use is best explored by studying closely related species or socially polymorphic species that differ in their social system, but share a common environment. Here we investigate whether five sympatric *Anelosimus* spider species that range from nearly solitary to highly social differ in their use of space and in their phenology as a function of their social system. By studying these species in Serra do Japi, Brazil, we find that the more social species, which form larger, longer–lived colonies, tend to live inside the forest, where sturdier, longer lasting vegetation is likely to offer better support for their nests. The less social species, which form single-family groups, in contrast, tend to occur on the forest edge where the vegetation is less robust. Within these two microhabitats, species with longer-lived colonies tend to occupy the potentially more stable positions closer to the core of the plants, while those with smaller and shorter-lived colonies build their nests towards the branch tips. The species further separate in their use of common habitat due to differences in the timing of their reproductive season. These patterns of habitat use suggest that the degree of sociality can enable otherwise similar species to differ from one another in ways that may facilitate their co-occurrence in a shared environment, a possibility that deserves further consideration.

## Introduction

Recent comparative studies of species or populations exhibiting different social behaviors have offered new insights into the ecological conditions that favor sociality (reviewed by [1]). In general, these investigations begin by identifying environmental factors, such as climatic variables, abundance of natural enemies, and resource availability, which correlate with natural variation in social traits (e.g. [2]–[9]). Some studies have then tested the impact of these environmental gradients by manipulating specific ecological factors [9], [10], or by transplanting organisms across environmental gradients [9], [11] or into common gardens [12]. Thus, we now know that many extrinsic factors may affect the costs and benefits of sociality, thereby shaping the distribution of social and less social species.

When comparing between social and non-social organisms in the same lineages (including Allodapine and Halictine bees [13], [14]; aphids [15]; thrips [16]; and social spiders [17], [18]), we can identify variation in a few key, inter-related traits that characterize the social categories. These traits, in turn, have been linked to some of the environmental factors listed above. (1) Dispersal behavior. In non-social species, every individual should disperse from the natal colony and found a new nest independently, whereas in social species, individuals may remain together for multiple generations. Differences in dispersal behavior can therefore result in variation in the length of time that a nest is used, being relatively short-lived in non-social species, and relatively long-lasting in social ones. In general, previous studies have focused on the risk of dispersal as a force favoring the formation of social groups. These risks can be higher, for instance, in arid environments, where dispersal can only occur during rare and unpredictable rainstorms (e.g. [16], [19]), or in habitats with greater predation pressure (e.g. [10]). (2) Group size. This characteristic is likely to interact with a range of ecological factors. For example, larger groups will require burrows or nests that are many times larger than those required by solitary organisms, which can impose constraints on the positions where social organisms can nest. On the other hand, social individuals may be better protected from predators by such simple mechanisms as the selfish herd effect [20]–[22]. (3) Cooperation. By working together, social organisms may be able to increase their efficiency relative to solitary individuals. For example, cooperation in nest maintenance or brood care may allow a larger workforce to focus on attaining food resources or defending against natural enemies (e.g. [23], [24]). Thus, a relatively straightforward shift in social behavior can result in a whole suite of differences in the way that organisms interact with their environment.

So far, most of these differences, both in the environmental factors that influence sociality and in the social traits themselves, have been observed and measured in allopatry [1]. Investigating the ecology of sociality in species living in sympatry, however, offers the possibility of exploring how such species differ from one another in a shared environment. Here, we investigate the ecological characteristics of five sympatric, congeneric spider species that range from almost solitary to highly social. We ask whether species differ in their use of space and time as a function of their social system, with the degree of sociality defined in this context by the size and duration of the nest. Based on earlier studies of differentiation between otherwise similar species that differ in body size (e.g. [25], [26]), we expect that the degree of sociality (i.e. differences in nest size and longevity) could contribute to differentiation in habitat use.

**Figure 1:**
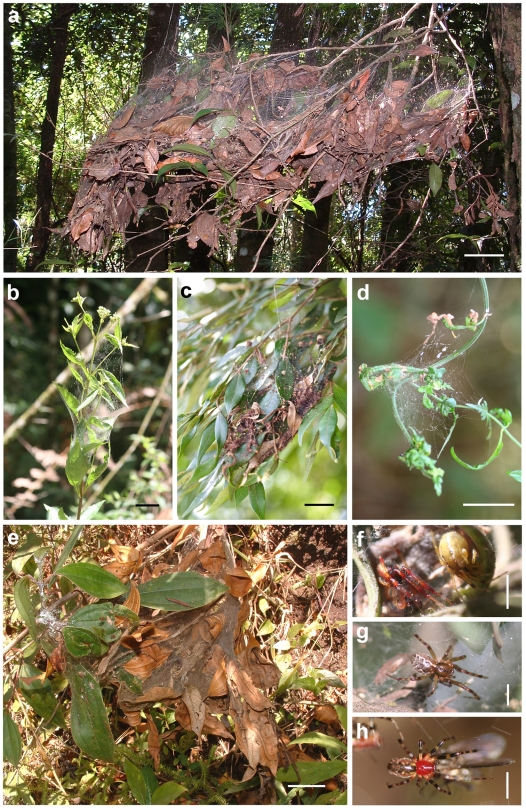
Photographs of the study organisms. Webs of *Anelosimus jabaquara* (A, scale bar 10 cm), *A. baeza* (B), *A. studiosus* (C), *A. nigrescens* (D), and *A. dubiosus* (E, B–E scale bar 5 cm). Also shown are male and female of *A. nigrescens* (F), female of *A. baeza* (G), and female of *A. jabaquara* (H, F-H scale bar 2.5 cm).

We first consider two alternative hypotheses: species exhibiting similar social traits could either occupy similar habitat types (e.g. open habitat vs. forest) due to similar resource or space requirements (H_1_) or segregate in separate environments to possibly avoid competing for the same resources (H_2_). The null hypothesis is that the species utilize space with no reference to their social system (or other shared characteristics) (H_0_). If H_1_ is supported, we further ask whether other habitat dimensions, at either other spatial scales or in time, contribute to the separation of similar species in niche space. If our results are consistent with H_2_, we further investigate how species with different degrees of social behavior differ in their utilization of shared resources. A complementary study investigated the dietary differences between these species [27].

## Methods

### Species descriptions

*Anelosimus* (Theridiidae, Araneae) species exhibit social behaviours ranging from nearly solitary to subsocial (non-territorial periodic social) and highly social (non-territorial permanent social) [23], [28]. Social species occupy a shared nest for multiple generations, where group members cooperate in brood rearing, prey capture, and nest maintenance. Depending on the species and habitat, these social spider nests may grow to contain hundreds to thousands of individuals. In contrast, the nests of subsocial species typically contain a single-family group, as adults usually nest solitarily and the offspring disperse prior to reaching reproductive maturity [23]. The period of cooperation and cohabitation by siblings may be shorter in some species, creating a continuum between solitary and subsocial strategies [29]. There is no evidence of any cooperation or even regular contact occurring between spiders from different nests in any members of this genus.

The five *Anelosimus* species that co-occur in Serra do Japi, Brazil differ from one another in their social system [30]. *Anelosimus dubiosus* Keyserling is the most social, as its colonies may last multiple generations without dispersing and new nests appear to be initiated by inseminated females dispersing alone or in small groups from the same source nest [31]. *Anelosimus jabaquara* Levi seems to be intermediate between social and subsocial since dispersal from the natal nest appears to be partial– some females remain to reproduce thus yielding colony sizes comparable to those of *A. dubiosus* [32]. These two species are found at the southern edge of the tropical zone in Brazil (∼20–25° S), where they often occur in sympatry ([28], [33] also MO Gonzaga, unpublished data).

**Figure 2:**
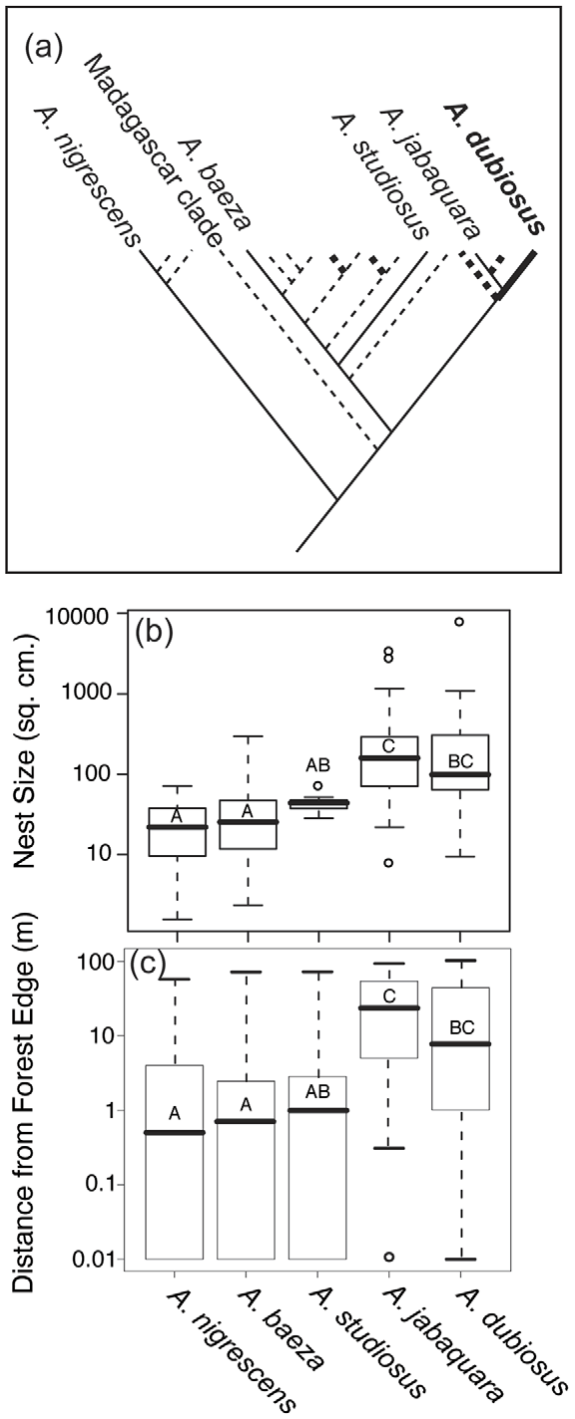
The phylogenetic relationship, nest size, and distance from forest edge are shown for the five focal species. The bold lines in the phylogeny diagram (a) represent highly social spider species (phylogeny redrawn from [33]). The five species that we observed in Serra do Japi, which were used in our analyses, are labeled in this figure. Other species are shown to demonstrate the phylogenetic distances between focal species. These species differ in the median nest size by an order of magnitude, with more social species building larger nests (b). The more social species tend to occur further from the forest edge than the subsocial and nearly solitary species (c) The boxplots show the median and the upper and lower quartiles. The whiskers encompass the 1.5x the interquartile range, and circles represent outliers. Letters show statistically significant differences between species (Dunnett-Tukey-Kramer test for b, posthoc test on Kruskal-Wallis for c).

*Anelosimus studiosus* Hentz and *Anelosimus baeza* Agnarsson are typical subsocial species, with dispersal occurring primarily at subadult instars each generation. Both species are distributed throughout South America in areas outside the lowland tropical rainforest (e.g., at higher elevations, higher latitudes, or drier habitats) [17], [28]. *Anelosimus studiosus* extends into North America where the more northern populations may form groups of multiple females and their offspring [3], [34]. The fifth species, *Anelosimus nigrescens* Keyserling, is considered nearly-solitary due to the early dispersal of immature individuals, the reduced maternal care phase, and elevated degree of aggression among siblings ([33], [35] also MO Gonzaga and J Vasconcellos-Neto, unpublished data). *Anelosimus nigrescens* is found in Brazil's Atlantic coastal forest, as well as in Guyana and possibly Venezuela ([36] also MO Gonzaga, unpublished data). All five species build irregular three-dimensional webs (Fig. 1), which are occupied for periods reflective of their degree of sociality–fractions of a generation, for the less social species, to multiple generations, for the more social ones. The webs are used both to intercept prey and to shelter the inhabitants from predators and from the elements. The phylogenetic relationships of these species are shown in Fig. 2a (reconstructed from [33]). A complementary study found that these four species differ in the size of the prey that they capture and consume, although there is some overlap. In general, species with larger nests capture larger prey, while species with smaller nests capture smaller prey. A full range of prey sizes was available throughout the habitat [27].

### Habitat description

Serra do Japi is a Brazilian protected area located between the latitudes 23°12'–23°22'S and longitudes 46°57'–47°05'W, comprising an area of about 354 Km^2^. The vegetation is composed mainly of semi-deciduous forest, markedly seasonal, with leaf fall occurring especially during the dry and relatively cool autumn and winter seasons (from April to September). The habitat is dominated by Myrtaceae, Lauraceae, Meliaceae, Caesalpinaceae, Mimosaceae, Euphorbiaceae and Fabaceae. Our study area was located from about 1000 m–1200 m altitude and covered about 3 km^2^. Rains are concentrated in the first months of the summer (from October to January) and annual precipitation is about 1350 mm in the region [37]–[39]. The protected area does not allow manipulation or destructive sampling, so we limited ourselves to observing and measuring existing colonies.

### Sampling

We surveyed the nests of the five *Anelosimus* species along six transects (200 m×5 m) in Serra do Japi in November, 2005. Each transect was initiated at a randomly selected colony using the T-squared sampling method [40]–six points were randomly chosen along the accessible roads or trails within the reserve; we then found the nearest colony (of any species) to that point. We initiated each transect at the nearest neighbour of the first colony, and proceeded along a randomly selected compass bearing. Having a starting point near a road (as opposed to anywhere on the map) helped ensure at least some representation of edge habitat, which was represented in all six transects in proportions ranging from 10 to 40%. The forest edge consisted of shrubby habitat along human created edges (roads, trails, and overgrown pastures) and along natural streams and swamps. The forest interior consisted of a closed-canopy forest of a height up to about 25 m, with a mix of trees and shrubs. We acknowledge that some of the forest interior habitat that we surveyed may have been influenced by the edge effect, although in our sample, the plants recorded as used and available for web construction by the spiders differed considerably between these two habitat types.

The nests of most *Anelosimus* spider species consist of three-dimensional baskets built with dense webbing. The area of the largest horizontal cross section of the basket is proportional to the number of individuals in a colony [41]. Above the basket, the “prey capture” area of the nest consists of a mesh of looser threads where most prey items are intercepted. Nests of some species, including *A. nigrescens* among the species investigated here, lack the basal basket, but rather build nests consisting entirely of the looser “prey capture” webbing. For each nest encountered, we measured two nest- and seven habitat-specific variables: the cross-section area of the nest basket and the prey capture webbing height, as well as the distance from the forest edge, canopy cover, forest height, vegetation substrate identity, vegetation substrate diameter at breast height, nest height above the ground, and the location of the nest on the plant (detailed variable descriptions listed in Table 1). We measured the same habitat variables at 20 randomly positioned points along each transect in order to assess the habitat available to the spiders. In December, 2010 we added 4 additional transects that followed parallel trajectories along the forest edge and the forest interior at two sites in Serra do Japi. At 20 random points along these transects, we measured plant height, diameter at 50 cm (knee) height, diameter at breast height, and the length and width of the longest branch available for the spiders to build on. These variables were combined to form a ‘vegetation sturdiness’ index to allow for more systematic comparisons between the forest edge and forest interior habitat (Table 1).

In the original transects, we collected voucher specimens from nests for which species identification was not possible in the field. These individuals were then reared until adulthood in the lab and identified. We also documented the instars of the spiders in each nest (Table 1). Because the protected area prohibited destructive sampling, we were not able to collect vouchers from some nests that were positioned high above the ground, and these colonies were not included in this analysis. This inability to identify the highest nests may have skewed our height above ground comparisons, since the highest nests were not included.

**Table 1:** A description of the variables measured in Serra do Japi.

Measurement	Description
Spider Nest Characteristics	
Nest Size	Area of the largest horizontal cross section of the nest basket; *A. nigrescens* sometimes has a less clearly defined nest basket, so we measured the longest and widest horizontal web cross section(Purcell and Avilés 2007)
Prey Capture Web Height	Greatest vertical extent of the loose webbing above the basket (Purcell and Avilés 2007)
Habitat-Scale Spatial Measurements	
Distance from Forest Edge	Distance from the nest to the nearest forest edge, measured up to 10 m and estimated at longer distances
Percent Canopy Cover	Visual estimate of the percent of canopy cover directly above the nest
Forest Height	An estimate of the average height of the canopy above the nest
Local-Scale Spatial Measurements	
Plant Identity (Substrate)	Identity of the plant supporting the nest (classified to family)
Height Above Ground	The distance from the lowest part of the nest's basket to the ground below it
Plant Diameter at Breast Height (DBH)	The size of the plant supporting the nest, classified in categories: 0: plants shorter than1.4 m; small: up to 10 cm diameter; medium: up to 40 cm; large: greater than 40 cm
Nest Position on Plant	Position of the nest on the plant: branch tip, middle of the branch, core of the plant
Vegetation sturdiness index	An index based on: plant height, DBH, diameter at 50 cm (knee) height, length and diameter of the longest branch. The index is the first axis of a principal component analysis (see Appendix S1).
Temporal Measurement	
Spider Instar	Most nests contained a single instar (juvenile 1–4, subadult, adult, egg sac present); when two instars were represented, we assigned the nest to the category representing the most common instar. For analyses, juvenile instars were grouped into one category.

We determined the spider nest characteristics, habitat- and local-scale spatial measurements (except vegetation sturdiness index) and temporal measurement for each spider nest along each transect. In addition, we measured the habitat- and local-scale spatial (except vegetation sturdiness index) variables at 20 randomly selected points along each transect in 2005. The vegetation sturdiness index was calculated based on measurements taken in 2010 at 20 randomly selected points along two sets of two parallel transects, one of each along the forest edge and the other inside the forest.

### Analysis

#### Overview of differences among species

In total, we analyzed the characteristics of 34 A. nigrescens, 58 A. baeza, 7 A. studiosus, 52 A. jabaquara, and 31 A. dubiosus nests. We performed a non-linear principal components analysis (PCA) on all nest size, habitat, and temporal variables except plant substrate identity (9 variables, Table 1) using the dudi.mix function in R 2.10. We omitted the plant substrate variable from this analysis, because we observed spider nests on 19 different plant families, and the individual treatment of each of these categories made the results of the PCA difficult to interpret. We calculated the relationships between all of the variables used in the principal components analysis (Table 2) and used ANOVA and the Dunnett-Tukey-Kramer (DTK) test to determine whether there were significant differences among the species along the first three principal component axes. The DTK test adjusts for unequal variances and unequal sample sizes.

#### Interspecific differences in habitat use

For the metrics that differed between species in the principal component analysis, we further investigated biologically relevant variables. Because our forest position metrics (distance from forest edge, forest height, and canopy cover) and nest size measurements (nest size and prey capture height) were highly correlated, we evaluated only distance from forest edge and nest size here. We used ANOVA and the DTK test to compare the nest size, and the Kruskal-Wallis test and a posthoc test (equivalent to the Tukey test) to compare the distance from the forest edge across the five species. In order to determine whether the habitat positions of the species were independent of phylogenetic relationships, we estimated the divergence time of the five species using published sequence data for cytochrome c oxidase subunit I and NADH dehydrogenase subunit I mitochondrial genes [33] using the neighbour joining method in Mega 4.1 [42]. We then compared the distance from forest edge among the five species based on their divergence time and nest size using phylogenetically independent contrasts in the ‘ape’ package in R 2.10.

#### Comparison of Forest Edge and Forest Interior Habitats

We calculated a vegetation sturdiness index from the variables measured along forest interior and forest edge transects using the first axis of a PC analysis, and calculated the difference between the two habitats using a Welch T-test. We also compared the distribution of Asteraceae and Myrtaceae relative to the distance from the forest edge using a Welch T-test.

#### Interspecific differences in nest position

For the local scale variables, we compared the species living within the same type of habitat (forest edge or forest interior). We used the Binomial test to determine whether plant substrates were used by each species more than expected based on their abundances, the Wilcoxon test for pairwise comparisons of nest height above the ground, and the Pearson Chi-squared test (in R 2.10) to investigate pairwise differences in nest position relative to plant substrate DBH and the nest location on the plant (branch tips, mid-branch or plant core/trunk).

#### Interspecific differences in phenology

We used the Pearson Chi-squared test (in R 2.10) to investigate pairwise differences in phenology; for this analysis, we used four life-cycle categories representing the most common instar: juvenile, subadult, adult, and adult with eggsac.

#### Species distribution versus null expectation

We tested whether each species differed in their spatial position from the null expectation by performing a permutation test on each PC axis. In R 2.10, we compared the sum-squared deviation of each group mean from the overall mean of the observed data with the results from 10,000 randomized permutations of the dataset. We also investigated how each species was distributed relative to the available habitat by performing a non-linear PCA on observed nest positions versus the possible nest positions quantified at each of 20 random points along every transect. We compared the first two PC axes using ANOVA and DTK tests.

#### Intraspecific variation

We explored whether there were any intraspecific patterns in the distribution of nest sizes relative to the habitat position (distance from forest edge) or nest height above ground using Pearson's correlation, or relative to the plant substrate DBH or the nest location on the substrate using ANOVA. We excluded A. studiosus from these intraspecific comparisons due to our small sample size. Statistical tests were carried out in R 2.10.

#### Corrections for Multiple Comparisons

In order to reduce the chances of committing type I errors in our analyses, we used the Holm-Bonferroni correction method to rank our statistical data and adjust the alpha for each of the 68 interspecific comparisons. Under these conditions, our functional threshold alpha value was approximately 0.002. Because this correction is conservative (18 comparisons with p<0.05 were considered non-significant with this method, but the false discovery rate for this study should be approximately 3–4 type I errors), we also discuss our marginally non-significant data in light of the power of each analysis. We treated the intraspecific data separately, and used the Holm-Bonferroni correction to adjust our alpha for the four comparisons performed on each species. All unspecified statistical analyses were performed in JMP 5.1 (SAS Institute, Cary, NC).

## Results

### Overview and interspecific differences in habitat use

**Figure 3:**
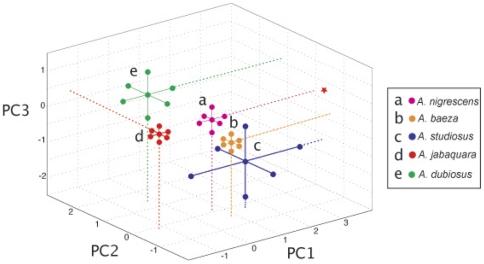
Three of the five species investigated show significant differences in their mean position along three principal component axes. The 95% confidence intervals for each species show moderate overlap between *Anelosimus studiosus* with *A. baeza* and *A. nigrescens,* but this may be due to the small sample size of *A. studiosus*. The social and intermediate social species (*A. dubiosus* and *A. jabaquara*) segregate from the subsocial and nearly solitary species (*A. studiosus, A. baeza* and *A. nigrescens*) along the first PC axis, which correlates with the distance from the forest edge (more negative values indicate that nests occur further inside the forest). Along the second PC axis, *A. dubiosus* differs from the other four species; more positive values indicate nests that are closer to the ground and built on the core of the plant. The three subsocial and nearly solitary species differ along the third PC axis, which reflects the local nest position and instar. The star represents the point where the confidence intervals measuring the position of *Anelosimus jabaquara* intercepts the y and z axis.

Overall, we found that species with large, long-lived nests (more social; Fig. 2) tended to occur in forested habitat characterized by sturdier vegetation consisting of larger plants with longer, thicker branches (vegetation comparison: t = −3.1, df = 82, p = 0.0027), while species with smaller, shorter lived nests (less social) tended to occur in more open, forest edge habitats on smaller, flimsier plants (ANOVA, PCA axis 1: F_(4, 172)_ = 32.9, p<0.0001; Figs. 2, 3). The nest sizes differed from one another by an order of magnitude (Fig. 2; F_(4, 177)_ = 34.2, p<0.0001), and species with larger nests built further inside the forest (Kruskal wallis test: χ^2^ = 57.69, DF = 4, p<0.0001). The phylogenetically independent contrast result suggests that this pattern cannot be explained solely by phylogenetic niche conservatism (F_(1, 3)_  = 17.63, p = 0.025).

**Table 2:** Associations between all variables used in the principal components analysis.^1^

Variable	Prey Capture Web Height	% Canopy Cover	Distance from Forest Edge	Forest Height Estimate	Nest Height Above Ground	Plant Diameter (ord)	Location on Plant (ord)	Instar (ord)
Nest Size (Cross Section Area)	Rho = 0.728, p<0.0001*	Rho = 0.362, p<0.0001 *	Rho = 0.517, p<0.0001 *	Rho = 0.489, p<0.0001*	Rho = −0.417, p<0.0001 *	χ^2^ = 5.69, p = 0.128	χ^2^ = 0.580, p = 0.748	χ^2^ = 22.6, p = 0.0009 *
Prey Capture Height	XXX	Rho = 0.221, p = 0.0028	Rho = 0.438, p<0.0001 *	Rho = 0.356, p<0.0001*	Rho = −0.307, p<0.0001*	χ^2^ = 0.878, p = 0.831	χ^2^ = 0.114, p = 0.945	χ^2^ = 11.8, p = 0.0659
% Canopy Cover		XXX	Rho = 0.608, p<0.0001 *	Rho = 0.658, p<0.0001 *	Rho = −0.107, p = 0.151	χ^2^ = 18.8, p = 0.0003 *	χ^2^ = 10.5, p = 0.0052	χ^2^ = 8.12, p = 0.230
Distance from Forest Edge			XXX	Rho = 0.743, p<0.0001*	Rho = −0.1638, p = 0.0267	χ^2^ = 10.9, p = 0.0124	χ^2^ = 7.14, p = 0.0281	χ^2^ = 16.6, p = 0.0111
Forest Height Estimate				XXX	Rho = −0.122, p = 0.101	χ^2^ = 19.5, p = 0.0002 *	χ^2^ = 21.0, p<0.000*	χ^2^ = 21.0, p = 0.0018
Nest Height Above Ground					XXX	χ^2^ = 35.3, p<0.0001 *	χ^2^ = 8.82, p = 0.0121	χ^2^ = 14.63, p = 0.0233
Plant Diameter (ord)						XXX	χ^2^ = 16.8, p = 0.010	χ^2^ = 37.3, p = 0.0048
Location on Plant (ord)							XXX	χ^2^ = 12.7, p = 0.39

1 Spearman's Rho correlation coefficients are used for the continuous variables, Kruskal-Wallis tests for continuous by ordinal comparisons, and Contingency tests for ordinal by ordinal comparisons. Stars indicate significant comparisons following the Holm-Bonferroni correction for multiple comparisons.

The highly correlated measures of nest size and position relative to the forest edge (Table 2) strongly influenced the first principal component axis (more negative values reflect larger nests that are positioned further inside the forest), which accounted for 22.5% of the observed variation in nest position (Fig. 3, Table 3). Species in similar environments also tended to build nests on the same type of plant. Along the forest edge, *A. baeza* nests were found on Asteraceae plants (Binomial test: p = 0.0009), and the nests of the other two species were also frequently found on Asters, although this trend was non-significant (*A. nigrescens*: p = 0.02; *A. studiosus*: p = 0.3). Inside the forest, the intermediate social species *A. jabaquara* was found on Myrtaceae plants more often than expected by chance (Binomial test: p = 0.0002), and *A. dubiosus* showed a similar non-significant trend (p = 0.07). The distribution of each plant type follows a similar pattern, with Asteraceae occurring nearer the forest edge than Myrtaceae, on average (Welch's T-test, t = 4.97, DF = 25, p<0.0001). These findings are consistent with our first hypothesis, that species with similar social systems share similar habitat requirements (H_1_).

**Table 3:** Principal components analysis results, indicating the weight of each variable on each PC axis as well as the eigenvalue and % of the variance accounted for by each axis.

	PC Axis			
Variable	1	2	3	4
Nest Size	−0.567	0.504	−0.458	0.331
Prey Capture Web Size	−0.622	0.510	−0.401	0.288
Distance From Forest Edge	−0.647	−0.042	0.149	−0.468
Canopy Cover	−0.677	−0.224	0.416	0.206
Forest Height	−0.829	−0.191	0.181	−0.116
Height Above Ground	0.160	−0.688	−0.522	0.074
DBH (Ord.L)	−0.352	−0.593	−0.145	0.297
DBH (Ord.Q)	0.107	0.142	0.507	0.462
Location on Plant (Ord. L)	−0.381	−0.350	−0.275	−0.310
Location on Plant (Ord. Q)	0.098	0.357	−0.291	−0.473
Instar (Ord.L)	−0.309	0.149	0.284	−0.319
Instar (Ord.Q)	0.123	0.247	0.073	−0.165
Eigenvalue	2.7	1.8	1.4	1.2
% Variance	22.5	15	12	10

The ordinal variables (DBH, location on plant, and instar) are converted to polynomials in this function, and the contribution of both components is shown.

### Interspecific differences in nest position

Inside the forest, we found that the social species *A. dubiosus*, with large nests that may be expected to remain intact through many generations (over the course of months or years), tended to occupy seemingly sturdier and longer-lasting nest positions than the intermediate social *A. jabaquara*. In general, *A. dubiosus* nests were found closer to the ground (χ^2^ = 9.65, p = 0.0019) and on shorter plants (χ^2^ = 62.5, p<0.0001) than *A. jabaquara*. Interestingly, *A. dubiosus* nests were also located on the core of the plant, while *A. jabaquara* nests were positioned closer to the tips of branches (χ^2^ = 228.0, p<0.0001). This difference was reflected in the positions of these species along principal component axis 2, where more positive values indicate nests that are closer to the ground and built on the core of the plant (F_(4, 172)_  = 12.1, p<0.0001; Fig. 3, Table 3).

**Figure 4:**
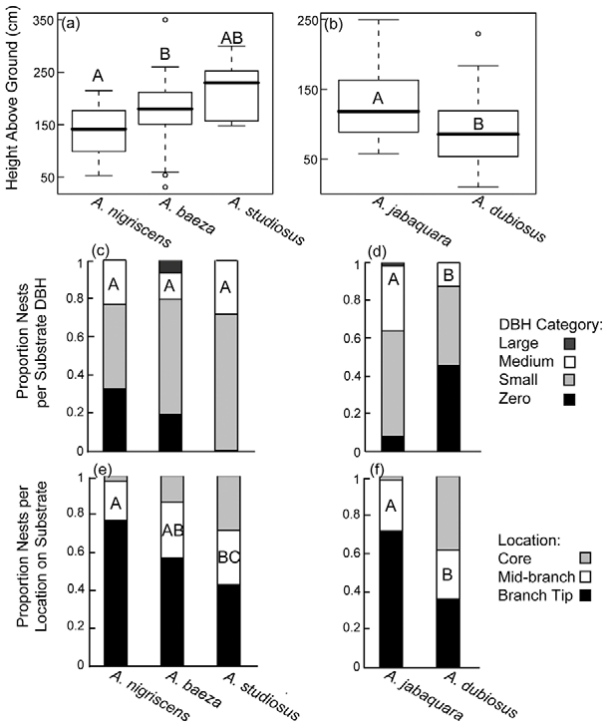
We compare local scale spatial variables, including nest height above ground, vegetation substrate diameter at breast height, and nest location on substrate. Comparisons of forest edge species (left panel) and forest interior species (right panel) show that species in both habitats show some differences in nest position. Significant differences between comparisons are shown with different letters.

Among the less social species found at the forest edge, we found more subtle differences in the nest position of each species (Figs. 3, 4). In this case, *A. nigrescens* tended to nest closer to the ground relative to *A. baeza* (χ^2^ = 13.2, p = 0.0003;) and *A. studiosus* (χ^2^  = 6.76, p = 0.0093), but the latter species did not differ from one another (χ^2^ = 1.54, DF = 1, p = 0.22). The nests of *A. nigrescens* also tended to be found more often on the branch tips than those of *A. baeza* (χ^2^ = 6.07, p = 0.048) and *A. studiosus* (χ^2^ = 13.2, p = 0.0003). Again, the two subsocial species did not differ from one another in nest position (χ^2^ = 1.35, p = 0.51), nor did we find any difference in the size of the plants used (Fig. 4, Appendix S1). These differences were reflected by differentiation along the third PC axis (Fig. 3; F_(4, 172)_ = 7.92, p<0.0001). The sample size for *A. studiosus* was small, however, so the comparison between the two subsocial species has little power.

### Interspecific differences in phenology

**Figure 5:**
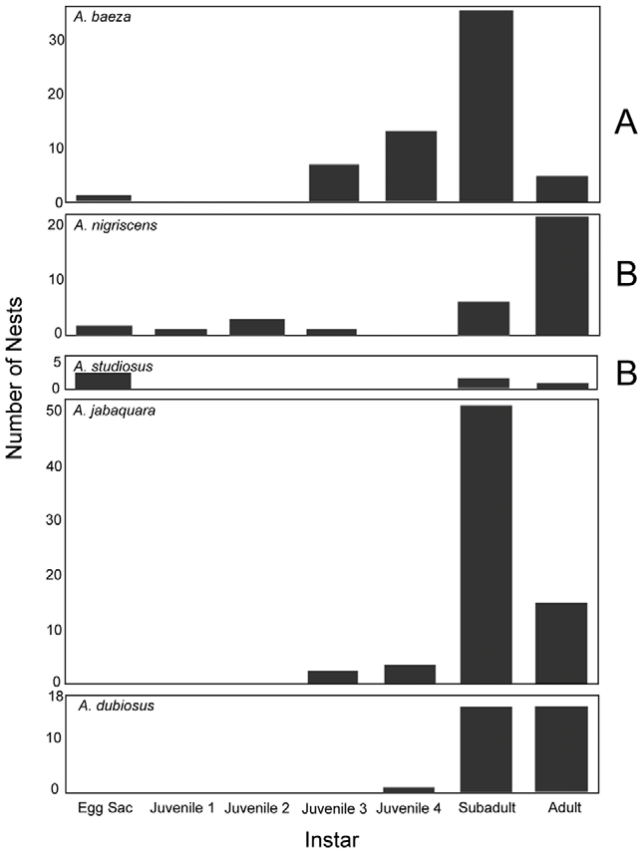
The bars show the most common instar present in each of the observed nests. Significant pairwise differences in age structure (Holm-Bonferroni corrected Chi-squared tests, with juveniles comprising a single category) are shown with letters to the right of each species diagram for the subsocial species. A previous long-term study found *Anelosimus jabaquara* and *A. dubiosus* to be offset by one month in their phenology [31], so we show the age structure of these species here for comparative purposes.

Among the three forest edge species, we also found a significant difference in the age structure of the colonies (Fig. 5). We found that the nearly solitary species *A. nigrescens* and the subsocial species *A. studiosus* were further advanced into their reproductive season at the time of our study than the subsocial *A. baeza*, as judged by the proportion of colonies containing adults and eggsacs (age structure difference: χ^2^ = 34.9, DF = 3, p<0.0001; χ^2^ = 23.6, DF = 3, p<0.0001, respectively). In spite of our small sample of *A. studiosus*, we found a marginally non-significant difference in the age structure of *A. nigrsescens* and *A. studiosus* (χ^2^ = 11.5, DF = 3, p = 0.0093); the former species seems to have a longer reproductive season, given the broad range of ages observed in nests during this two week observation period (Fig. 5). Inside the forest, a previous study found that the more social species, *A. jabaquara* and *A. dubiosus*, differ in their phenology by about one month [31], which is consistent with our qualitative result that a higher proportion of *A. dubiosus* nests contained adults (Fig. 5).

### Species differences versus null expectation

We were able to reject our null hypothesis (H_0_) that species utilize space without reference to their social system, as the distribution of these species differed from our null model (permutation test, PC axis 1: p<0.0001; PC axis 2: p = 0.003; the results from the PC axis 2 comparison were marginally non-significant with Holm-Bonferroni correction) and from the null expectation given our measure of available habitat (PCA, axis 1: F_(5, 303)_ = 16.9, p<0.0001; see Appendix S1). We also found that the position of *A. dubiosus* nests on the substrate (both the height of nests and the position of nests on branches or plant core) did not differ from the random expectation with respect to available nest positions; *A. jabaquara* and the three forest edge species, in contrast, tended to build nests on taller plants and more toward the branch tips than would be expected by chance (PCA, axis 2: F_(5, 303)_  = 14.4, p<0.0001).

### Intraspecific variation

Within species, we found a positive correlation between nest size and distance from the forest edge, although this correlation was only significant in *A. nigrescens* (Pearson's correlation r = 0.47, df = 32, p = 0.0054; other species: *A. baeza* r = 0.23, df = 56, p = 0.086; *A. jabaquara* r = 0.18, df  =  50, p = 0.20; *A. dubiosus* r = 0.090, df = 29, p = 0.63). Larger nests of species *A. baeza* and *A. jabaquara* were built lower (closer to the ground) than small ones, although these correlations were not significant following corrections for multiple comparisons (*A. nigrescens* r = −0.19, df = 32, p = 0.28; *A. baeza* r  = −0.26, df = 56, p = 0.050; *A. jabaquara* r = −0.32, df = 50, p = 0.022; *A. dubiosus* r = −0.095, df = 29, p = 0.61). There were also some subtle relationships between nest size and the nest position and plant substrate DBH in *A. baeza* and *A. jabaquara.* In particular, larger *A. jabaquara* nests tended to occur on larger plants (F_(3, 48)_ = 3.77, p = 0.017). Similarly, small *A. baeza* nests tended to be found on slender plants, although there were larger nests both on plants with larger DBH and short plants that did not reach breast height (F_(3, 54)_ = 4.11, p = 0.011). Neither *A. dubiosus* nor *A. nigrescens* showed a significant pattern in nest size relative to plant subsrate DBH (F_(2, 28)_  = 1.25, p = 0.30; F_(2, 31)_  = 1.34, p = 0.28, respectively), and no species exhibited a significant pattern in size of nests relative to their position on the plants following Holm-Bonferroni correction, although the largest *A. baeza* nests tended to occur on mid-branch locations (*A. baeza* F_(2, 55)_  = 3.23, p = 0.047; *A. nigrescens* F = 0.17; p = 0.85; *A. jabaquara* F_(2, 49)_ = 1.76, p = 0.18; *A. dubiosus* F_(2, 28)_  = 0.48, p = 0.62).

## Discussion

Social species differ from their non-social relatives in a number of basic traits, including dispersal propensity (and, by extension, nest longevity), group size, and level of cooperation among group members. We are interested in how these differences might impact the way species interact with their environments and the potential role they may play in determining the species composition of a given guild. Studies that have investigated ecological differences in habitats where just one social system occurs have led to useful insights regarding the ecological factors that may be responsible for the evolution and maintenance of sociality. How sociality influences habitat use, on the other hand, is more effectively studied by looking at closely related species or socially polymorphic species that differ in their social system, but occupy a shared environment. Here, we explore differences in habitat use among five congeneric and sympatric species of spiders that range in social behavior from nearly solitary to fully social. We ask whether their degree of sociality might be an important axis that allows otherwise similar species to differentiate from one another in their shared environment.

We found that the five sympatric *Anelosimus* spider species at Serra do Japi varied in their nest sizes by an order of magnitude overall (Fig. 2). We observed differentiation in the micro-habitats these species occupied, which correlated with their level of sociality along several spatial axes (Fig. 3). We found that the two most so cial species, with larger nests and colonies (*A. dubiosus* and *A. jabaquara*), tended to be further inside the forest where their nests can occupy sturdier vegetation, while the three less social species with smaller nests (*A. nigrescens*, *A. baeza* and *A. studiosus*) were generally found at the forest edge where plant substrates were less robust (Fig. 2). Within each microhabitat, the species differed from one another in the average height above ground their nests occupied and on their positions relative to the core of the plant, with the differences being more marked between the two forest interior species (Fig. 4). The forest edge species also differed in their phenology (Fig. 5), and a previous study demonstrated phenological differences between the two forest interior species [31].

The finding that species closer on the sociality scale occupy similar habitat types is consistent with a process of habitat filtering [43], where species exhibiting similar functional traits tend to occur together in shared environments. In this case, the functional differences may emerge from the different nest construction needs of social *versus* subsocial species in this system. More social species may require sturdier plants to support their larger, longer-lived nests and the presence of branches above the nest to allow the construction of enough prey capture webbing to support the greater number of individuals in the colonies [44]. Plants were indeed larger and with longer and sturdier branches inside the forest than at the forest edge (Appendix S1). The distribution of social and less social species may also simply parallel the distribution of their preferred plant substrate; the Asters used by the subsocial and nearly solitary species tended to occur on the forest edge while Myrtaceae used by the more social species occurred inside the forest. Alternatively, the five species differ in how phylogenetically close they are to each other (Fig. 2a), so phylogenetic niche conservatism could shape their distribution. In the case of the distance from forest edge measure, however, our phylogenetically independent contrast suggests differences among species (based on nest size as a proxy for degree of sociality) cannot be explained by phylogenetic relationships alone. Moreover, we observe that even within species, larger nests tend to be found further inside the forest, which suggests that the forest interior may be a more suitable habitat for the species that have larger nests on average.

At a more local scale, our data are consistent with our second hypothesis that similar species should segregate from one another to avoid competition (H_2_). Both on the forest edge and inside the forest, species differed both in the height of their nests above the ground and in the position of their nests on the plant substrate (Fig. 4). This pattern again lends support to the idea that the more social species may require sturdier nest positions, since the longest-lived nests in each environments tended to be found on the core of the plants, while the species that occupy more ephemeral nests were found towards the branch tips. This pattern could emerge either actively or passively, with longer-lived nests requiring more robust nest positions, and shorter-lived nests able to persist on a wider range of substrates. Even within species, larger nests tend to be closer to the ground and built on larger substrate, suggesting that nest construction requirements contribute to the interspecific pattern. Alternatively, given the fact that all species build nests in a range of positions, it is possible that certain nest positions are better than others for all species, and that the interspecific differences that we observe result primarily from competition for nest sites.

Differences in phenology among forest edge species (Fig. 5) and forest interior species [31] may allow the species to disperse and rear young at different times of the year. Interestingly, the two species with the greatest age structure difference–*A. baeza* and *A. studiosus* (Fig. 5)–are also the two most similar in their social phenotype. The species with the intermediate phenology, *A. nigrescens*, is the least social, with dispersal occurring during the early juvenile stage (MO Gonzaga, unpublished data). In addition to the phenological difference between the forest interior species [31], *A. jabaquara* tend to disperse prior to sexual maturity, while *A. dubiosus* dispersers are solitary or small groups of gravid females. We speculate that differences in the timing and mode of dispersal can potentially reduce competition for nest sites and prey. A concurrent study found that nest size strongly influences the size of prey captured, so these species may differ in the timing of each nest stage, thereby partitioning prey sizes during some key stages of their life cycle [27].

Further study is needed to disentangle the mechanisms that contribute to the observed patterns in this system [45]. So far, our observations are based on correlative evidence, so many other factors, such as uncharacterized behavioural differences or potential differences in the physiological requirements of each species, could contribute to the observed pattern of nest distribution. In order to disentangle the relative contribution of degree of sociality from other species characteristics, we plan to expand this comparison to other species and to other habitats (manipulation was not permitted in Serra do Japi, but would open up many interesting possibilities in other habitats with sympatric species). Based on the observed distribution patterns, we speculate that habitat filtering due to the structural requirements of different nest sizes may be the dominant mechanism separating the more and the less social species between the forest edge and interior habitats. Within each habitat, the segregation of co-occurring species may reflect either competition for nest sites, leading to character displacement (a possibility for the two closely related forest interior species), or the assemblage of this community, coincidentally or otherwise, with species with differing microhabitat requirements (a possibility for the phylogenetically more distant forest edge species). Although nest sites may seem unlimited, the use of specific plant types, nest architectural constraints, high nest densities for this genus (0.055 nests/m^2^, or roughly one nest per 18 m^2^), and possible competition with other web-building spiders could reduce the number of effective nest sites. Competition for optimal web placement may in turn lead to character displacement in web position and the timing of dispersal, as Herberstein [46] observed in Linyphiid spiders. Alternatively, these particular species may have been assembled due to pre-exisiting microhabitat preferences and substrate requirements, resulting in species that are unlikely to compete for the same positions on a plant. Many arthropods are known to seek nesting sites with specific abiotic conditions (e.g. [47]–[49]). If each species were adapted to subtly different microclimatic conditions or had different substrate requirements based on the typical size and longevity of their nests this could drive or contribute to the observed patterns.

More broadly, traits associated with differences in the degree of sociality may contribute to guild assemblages in other organisms, although this idea has not been explicitly explored in many empirical systems (but see [27]). Ant community assemblages, for example, are often assessed through placement of baits, where species are ranked in a dominance hierarchy. In general, dominant species are not found to co-occur in shared habitat, but frequently overlap with subordinate species (e.g. [50]). Competitive species often exhibit large colony sizes with rapid recruitment and/or major workers [51], so variation in socially important traits may also mediate these assemblages. Nest size differences and nest structural requirements may also shape the membership of ant (and other social insect) assemblages, but this possibility has not been extensively investigated due to the challenge of assessing subterranean nests (but see [52]). Differences in the level of sociality may be even more important in organisms exhibiting a broader range of social systems similar to what we observed in the spiders, including wasps and bees, aphids, thrips, as well as many bird and mammal taxa. Even in microbes, the diversity of assemblages has been proposed as a factor that could increase the potential for evolution of cooperative behavior in some species under some conditions [53].

### Conclusion

We have presented evidence that sympatric social and subsocial *Anelosimus* spiders in Serra do Japi, Brazil exhibit differences in their use of spatio-temporal resources. We believe that this study provides an important first step that can be further pursued to improve our understanding of the way that social structure impacts how individuals or colonies interact with their environment. In this case, we have found that the degree of sociality may have important consequences for the nest construction requirements (reflected in the vegetation substrate and the position of the nests) of these spider species. Such functional differences, in turn, may facilitate the coexistence of otherwise similar species whose social structure enables them to differentiate in their diet [27], their habitat requirements, and/or the timing of key life cycle events.

## Supporting Information

Appendix S1 **Principal components analyses summaries and comparisons of species at each individual variable.**
(DOC)
