# Supplementary material for: Spatio-Temporal Differentiation and Sociality in Spiders
Source: PLoS One. 2012 Apr 24;7(4):e34592. doi: 10.1371/journal.pone.0034592 (PMC3335849; doi:10.1371/journal.pone.0034592)
Supplement: Appendix S1 — Principal components analyses summaries and comparisons of species at each individual variable. (DOC) [file pone.0034592.s001.doc]

Appendix S1. Principal components analyses summaries and comparisons of species at each individual variable.

We used principal components analyses to explore the relationships between our measurement of spider nests, habitat, and phenology. We found that the three variables that distinguished forest edge and forest interior habitat influenced our first principal components axis (Fig. S1) and were all positively correlated with one another (Table 2). Our measurements of local nest positions, including the height of the nest, the size of the plant used, and the location of the nest on the plant, influenced the second principal components axis. Our two measures of nest size, including the area of the largest horizontal nest cross section and the height of the prey capture web, influenced both the first and second principal components axes. The age of the spiders in the nests had a similar, though lesser, influence on both axes (Fig. S1). We found similar results for our comparison of the five species *versus* the available habitat and nest positions (Fig. S2). Overall, the first PC axis was influenced primarily by the variables that distinguish forest edge from forest interior (Table S1). In general, *A. jabaquara* tended to occur further inside the forest, and *A. nigrescens* and *A. baeza* closer to the forest edge than would be expected by chance (Fig. S2). Along the second axis, all of the species except *A. dubiosus* tend to be found towards the tips of branches and on larger plants than would be expected based on the available substrate (Fig. S2).

In our correlation matrix, we found that plant size tended to be greater inside the forest (Table 2). In order to quantify this difference, we added two sets of parallel transects (one inside the forest and one along the forest edge); along each transect, we measured 5 metrics of plant sturdiness at 20 random points (see methods). We then performed a PC analysis (Table S2) on these data to estimate a ‘vegetation sturdiness index.’ Lower values along the first PC axis correlated with taller, wider plants with longer, broader branches (Table S2; individual metrics shown in Fig. S3).

We then examined differences between species for each individual variable that we measured. We show that the difference in nest size across the five species (Fig. 2b) is also reflected in the difference in prey capture web height (Fig. S4a). We then compare the habitat position of each of the five *Anelosimus* species (Fig. S4). Overall, the social species *A. dubiosus* and the intermediate social *A. jabaquara* tended to occur further from the forest edge in habitat characterized by a more closed canopy (Fig. S4b) and taller trees (Fig. S4c; Table S3). In contrast, the two subsocial species (*A. studiosus* and *A. baeza*), as well as the nearly solitary *A. nigrescens* tended to live in relatively open habitat along the forest edge (Fig. S4, Table S3). As stated in the main text, these two habitats differed in the size and durability of the plants upon which *Anelosimus* spiders could build nests.

Within each type of habitat, spiders differed in the height of their nests above the ground and the positions of their nests on the substrate (Fig. 4). Inside the forest, *A. dubiosus* build nests on smaller plants than *A. jabaquara*, but we found no difference in the size of plants used by the three forest edge species (*A. nigrescens versus: A. baeza* χ^2^=9.38, p=0.025; *A. studiosus* χ^2^=3.53,

p=0.32; *A. baeza versus A. studiosus* χ^2^=3.06, p=0.38). This lack of differentiation may reflect the smaller range of plant sizes present along the forest edge.


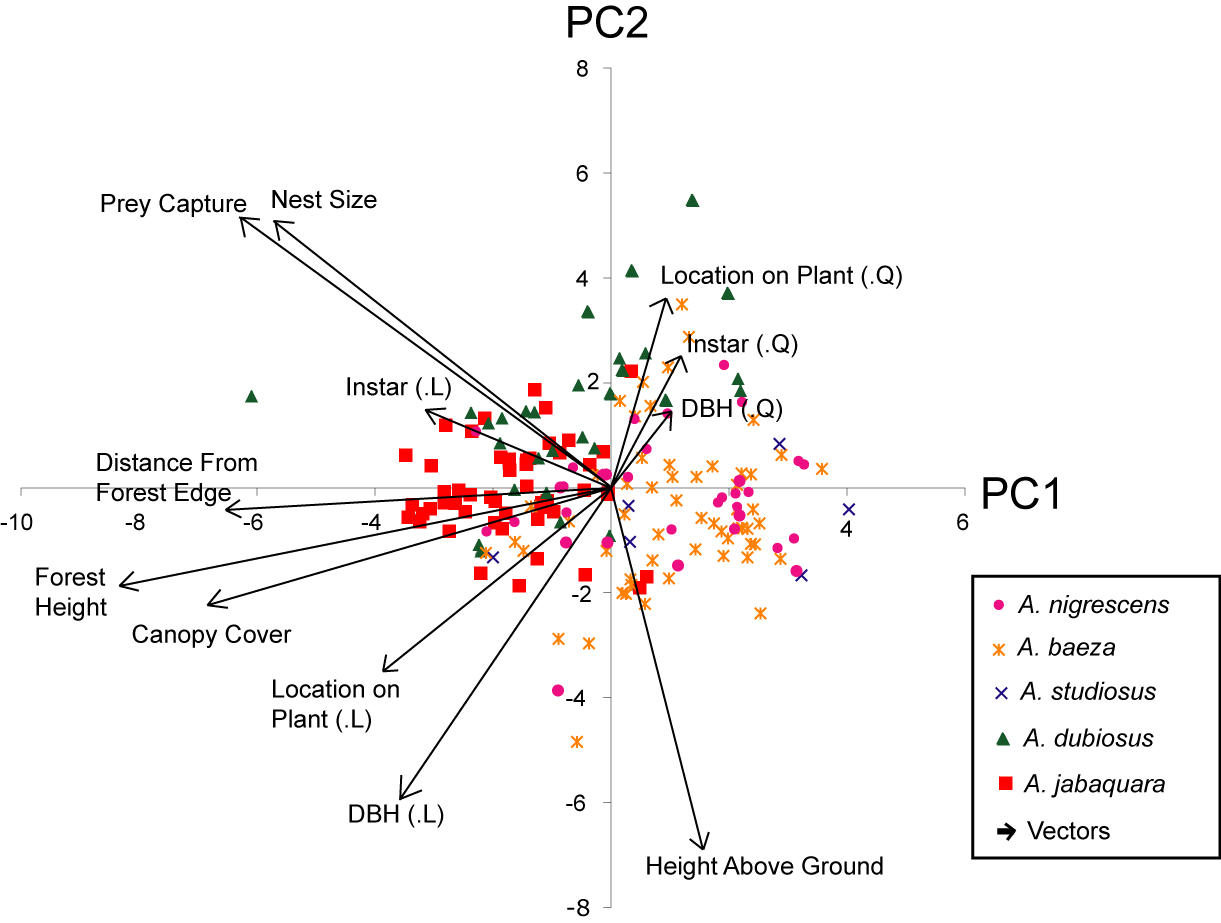


Figure S1: Position of individual colonies of each species along principal components axes 1 and 2. The colors match those shown in figure 3 of the text. The influence of each variable along each principal components axis is shown with a labeled vector. We contend that variables that differentiate forest edge and forest interior habitat, forest height, % canopy cover, and distance from forest edge, as well as nest size and prey capture height variables, are the most influential for principal component axis 1. Principal component axis 2 appears to be most influenced the local spatial scale variables, nest height above ground, plant diameter, and location on plant.


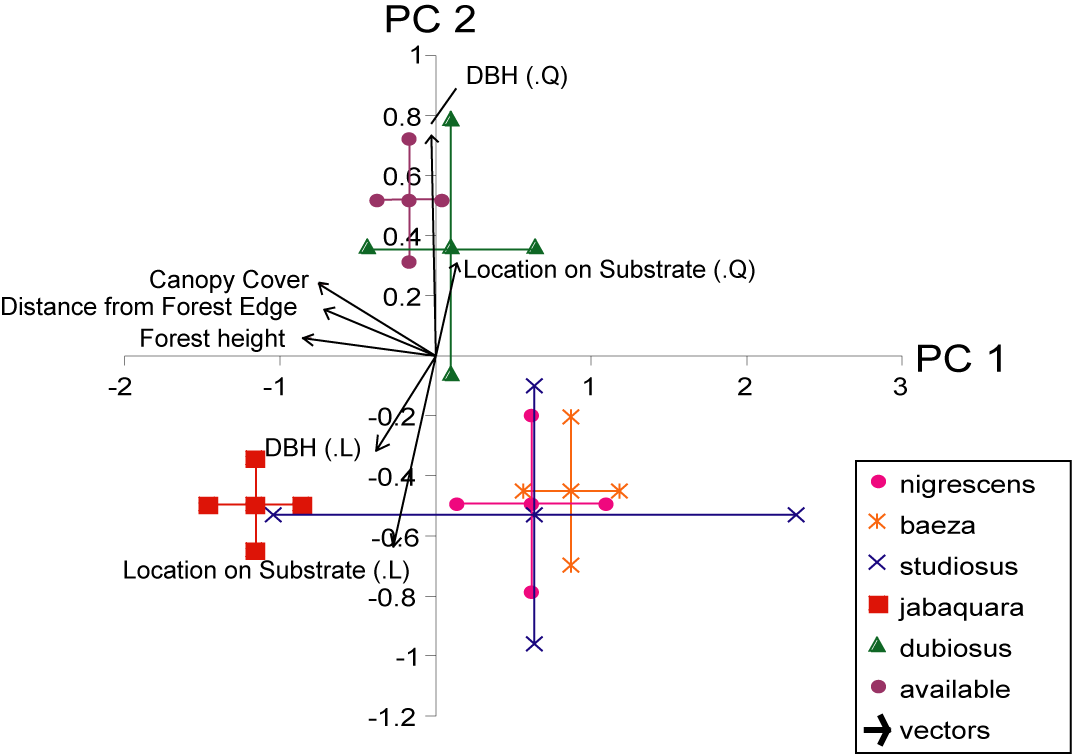


Figure S2: Mean +/- 95% CI position of each species *versus* the available habitat along principal components axes 1 and 2. The colors match those shown in figs. 3, S1. The influence of each variable along each principal components axis is shown with a labeled vector.

Table S1: Principal components analysis results for the comparison between *Anelosimus* species *versus* available habitat.

|  |  | PC Axis |  |
| --- | --- | --- | --- |
| Variable | 1 | 2 | 3 |
| Forest Height | -0.842 | 0.054 | -0.103 |
| Canopy Cover | -0.743 | 0.234 | 0.115 |
| Distance from Forest Edge | -0.695 | 0.147 | -0.339 |
| DBH (Ord.L) | -0.375 | -0.322 | 0.542 |
| DBH (Ord.Q) | -0.017 | 0.729 | 0.328 |
| Location on Plant (Ord.L) | -0.265 | -0.641 | -0.289 |
| Location on Plant (Ord.Q) | 0.144 | 0.304 | -0.729 |
| Eigenvalue | 1.976 | 1.218 | 1.156 |
| % Variance | 28% | 17% | 17% |

Table S2: Principal components analysis results for the vegetation sturdiness index. We used the first PC axis, which represents 52% of the observed variance, as the index. Along this axis, larger plants with longer, thicker branches have lower (negative) values.

|  |  | PC Axis |  |
| --- | --- | --- | --- |
| Variable | 1 | 2 | 3 |
| Plant Height | -0.486 | -0.354 | 0.799 |
| DBH | -0.824 | -0.431 | -0.288 |
| DKH | -0.863 | -0.366 | -0.257 |
| Longest Branch Length | -0.664 | 0.639 | 0.057 |
| Longest Branch Diameter | -0.698 | 0.599 | 0.047 |
| Eigenvalue | 2.587 | 1.212 | 0.793 |
| % Variance | 52% | 24% | 16% |


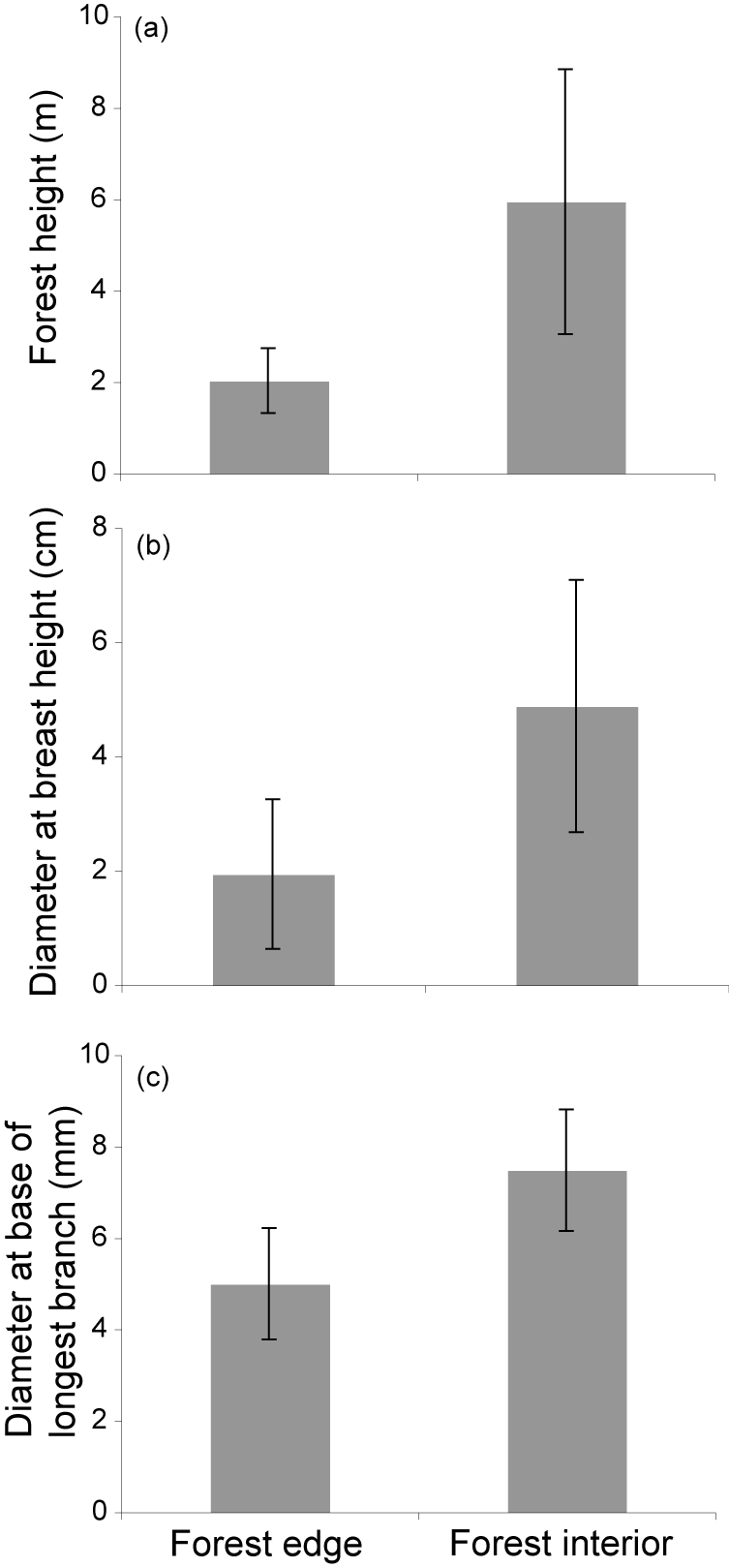


Figure S3: Along two sets of parallel transects, we found that random plants on the forest edge tended to be shorter (a) and smaller in diameter (b) than plants inside the forest. Moreover, the longest branches tend to be wider at their bases on plants inside the forest compared to those outside the forest (c). Figures show mean values and 95% confidence intervals.


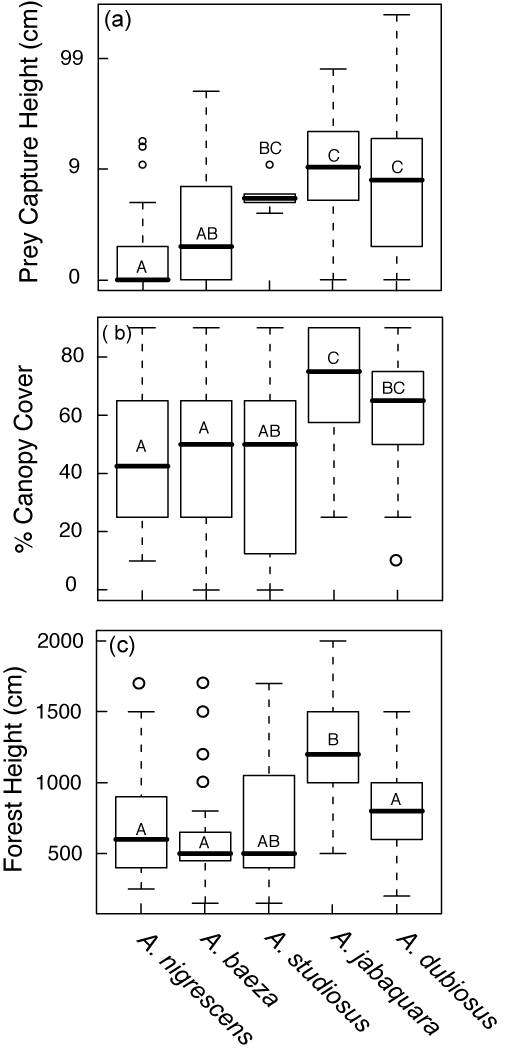


Figure S4: Comparisons of prey capture web height (the greatest vertical extent of the loose webbing above the nest basket; a), percent canopy cover (a visual estimate of the percent of canopy cover directly above the nest; b) and forest height (an estimate of the average height of the canopy above the nest; c) between the species. Letters show significant differences (see Table S3).

Table S3: Summary of pairwise statistical comparisons (Wilcoxon tests, alpha was corrected for multiple comparisons for differences reported in Fig. S4). Species are either compared with each other or with available habitat measures.

| Species | Comparison | Prey Capture Web Height  (Wilcoxon) | Canopy Cover (Wilcoxon) | Forest Height (Wilcoxon) |
| --- | --- | --- | --- | --- |
| *A. nigrescens* | *A. baeza* | χ^2^=5.85, p=0.016 | χ^2^=0.17, p=0.68 | χ^2^=0.33, p=0.57 |
|  | *A. studiosus* | χ^2^=12.2, p=0.0005 | χ^2^=0.061, p=0.81 | χ^2^=0.044, p=0.83 |
|  | *A. jabaquara* | χ^2^=32.2, p<0.0001 | χ^2^=19.6, p<0.0001 | χ^2^=22.3, p<0.0001 |
|  | *A. dubiosus* | χ^2^=18.7, p<0.0001 | χ^2^=6.17, p=0.013 | χ^2^=1.96, p=0.16 |
|  | Available | - | Less than null expectation  χ^2^=12.2, p=0.0005 | Less than null expectation  χ^2^=11.0, p=0.0009 |
| *A. baeza* | *A. studiosus* | χ^2^=2.87, p=0.09 | χ^2^=0.11, p=0.73 | χ^2^=0.0018, p=0.97 |
|  | *A. jabaquara* | χ^2^=24.1, p<0.0001 | χ^2^=24.9, p<0.0001 | χ^2^=47.4, p<0.0001 |
|  | *A. dubiosus* | χ^2^=8.56, p=0.0034 | χ^2^=6.93, p=0.0085 | χ^2^=9.17, p=0.0025 |
|  | Available | - | Less than null expectation  χ^2^=15.0, p=0.0001 | Less than null expectation  χ^2^=33.4, p<0.0001 |
| *A. studiosus* | *A. jabaquara* | χ^2^=2.56, p=0.11 | χ^2^=4.79,p=0.029 | χ^2^=4.15, p=0.042 |
|  | *A. dubiosus* | χ^2^=0.41, p=0.52 | χ^2^=2.1, p=0.15 | χ^2^=1.05, p=0.31 |
|  | Available | - | χ^2^=1.80, p=0.18 | χ^2^=2.37, p=0.12 |
| *A. jabaquara* | *A. dubiosus* | χ^2^=1.64, p=0.20 | χ^2^=2.97, p=0.085 | χ^2^=13.0, p=0.0003 |
|  | Available | - | Greater than null expectation  χ^2^=9.35, p=0.0022 | Greater than null expectation  χ^2^=17.7, p<0.0001 |
| *A. dubiosus* | Available | - | χ^2^=0.27, p=0.60 | χ^2^=1.65, p=0.20 |
